# Regulation of NK-Cell Function by HLA Class II

**DOI:** 10.3389/fcimb.2020.00055

**Published:** 2020-02-18

**Authors:** Annika Niehrs, Marcus Altfeld

**Affiliations:** ^1^Research Department Virus Immunology, Heinrich Pette Institute, Leibniz Institute for Experimental Virology, Hamburg, Germany; ^2^Institute for Immunology, Universitätsklinikum Hamburg Eppendorf, Hamburg, Germany

**Keywords:** natural killer cells, HLA class II, HLA-DP, immune cross-talk, HBV

## Abstract

Natural Killer (NK) cells were initially described as part of the innate immune system and characterized by their ability to lyse malignant and virus-infected cells. The cytolytic function of NK cells is tightly controlled by activating and inhibitory receptors expressed on the cell surface. Ligands that interact with a variety of NK-cell receptors include the human leukocyte antigen (HLA) molecules, and the regulation of NK-cell function by HLA class I molecules is well-established. Earlier studies also suggested a role of HLA class II molecules in regulating NK cell activity; yet, interactions between HLA class II molecules and NK cell receptors have not been well-characterized. We recently identified a subset of HLA-DP molecules that can serve as ligands for the natural cytotoxicity receptor NKp44 and activate NK cells. This novel receptor-ligand interaction provides a potential mechanism to explain the strong associations of HLA-DP molecules with HBV infection outcomes, graft-vs.-host disease and inflammatory bowel disease. Furthermore, it adds a new mechanism for NK-cell crosstalk with immune cells expressing HLA class II molecules. In this perspective article, we discuss the potential implications of NK cell receptor interactions with HLA class II molecules for the regulation of immune responses.

## HLA Class II Molecules Can Serve as Ligands for NK Cell Receptors

The functional activity of Natural Killer (NK) cells is regulated by the expression of inhibitory and activating receptors, many of which interact with human leukocyte antigen (HLA) molecules. HLA class I (HLA-I) molecules have been well-characterized as ligands for the NK cell receptor group of killer-cell immunoglobulin like receptors (KIR) (Jost and Altfeld, 2013). The specificity of KIR-HLA-I interactions as well as the influence of peptides presented by HLA-I on KIR-binding has been extensively studied (Vales-Gomez et al., 1998; Moesta et al., 2008; Fadda et al., 2011; Rahim et al., 2014; Guethlein et al., 2015; Holzemer et al., 2015; O'Connor et al., 2015; Garcia-Beltran et al., 2016; Chapel et al., 2017; Naiyer et al., 2017). HLA-I complexes consist of a polymorphic α-chain and a conserved chain, termed β2-microglobulin, and present intracellularly-derived peptides on the cell surface. HLA-I molecules are expressed on the surface of all nucleated cells. In contrast, the expression pattern of HLA class II molecules (HLA-II) is mainly restricted to antigen-presenting cells under homeostatic conditions (Muhlethaler-Mottet et al., 1997; Ting and Trowsdale, 2002). However, also non-hematopoietic cells have been shown to express HLA-II molecules after exposure to IFN-γ (Herkel et al., 2003; Stevanovic et al., 2013). HLA-II molecules consist of two polymorphic chains, a α- and a β-chain, and mainly present exogenous-derived peptides. Surface-expressed HLA-II complexes classically interact with CD4^+^ T cells; however HLA-II recognition has also been described for CD8^+^ T cells, especially in the context of chronic virus infections (Heemskerk et al., 2001; Rist et al., 2009; Ranasinghe et al., 2016). Earlier studies suggested a regulation of NK cell activity not only by HLA-I but also HLA-II molecules (Jiang et al., 1996; Lobo et al., 1996). In particular, reduced cytolytic activity of NK cells has been reported after co-incubation with HLA-DR^+^ target cell lines in comparison to non-HLA-II-expressing target cell lines (Jiang et al., 1996). Which NK cell receptors are involved in the recognition of HLA-DR and subsequent inhibition of NK cells remains unknown. The authors suggested a “missing-self” hypothesis not only for HLA-I but also HLA-II molecules and further discussed the possibility of specific NK cell populations not only recognizing HLA-DR, but also HLA-DQ and HLA-DP molecules (Jiang et al., 1996).

**Figure 1:**
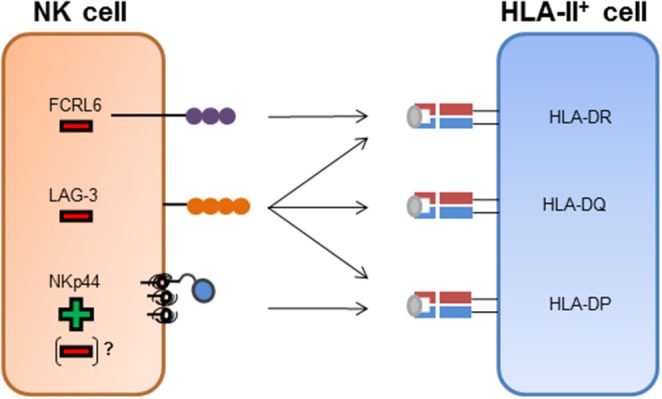
NK cell receptor interactions with HLA class II molecules. FCRL6^+^ NK cells have been shown to interact with HLA-DR molecules. The binding of FCRL6 to HLA-DR molecules inhibits NK cell function. LAG-3 has been described to bind to HLA-II molecules and has been attributed an inhibitory function after engagement of HLA-II molecules. NKp44 has been described to bind to a subset of HLA-DP molecules and transmit activating signals after binding. Inhibitory splice isoforms of NKp44 expressed on tissue-resident NK cells or NKp44 expression on other innate immune cells might transmit inhibitory signaling after engagement of HLA-DP molecules.

We recently identified a subset of HLA-DP molecules as ligands for the activating NK cell receptor NKp44 (Figure 1). The interaction between NKp44 and HLA-DP was dependent on the HLA-DP allotype and further modulated by the peptide presented by HLA-DP molecules (Niehrs et al., 2019), reminiscent of KIR binding to HLA-I. KIR molecules have been crystallized in complex with HLA-I molecules, and these structures clarified how KIR-HLA-I interactions are modulated by the presented peptide, in particular by the C-terminal amino acids of the peptide (Maenaka et al., 1999; Boyington et al., 2000; Fan et al., 2001; Liu et al., 2014). NKp44 has not yet been crystallized in a ligand-bound state and a crystal structure of NKp44 in complex with HLA-DP will help to further elucidate the factors that determine binding of NKp44 to HLA-DP. In contrast to previous studies, we detected activation of NK cells after NKp44-binding to HLA-II. Since we observed differential binding of HLA-DP to NKp44 in a peptide-dependent manner, we cannot exclude that NKp44 is able to bind to other HLA-II molecules during malignancies or infection, where these molecules present a different peptide reservoir. NKp44 has been described as an activating NK cell receptor, but also has an inhibitory splice isoform, NKp44-1 (Cantoni et al., 1999). This inhibitory splice form of NKp44 is the predominant form in decidua tissue (Siewiera et al., 2015), and engagement of HLA-DP molecules by tissue-resident NK cells might result in a different functional activity than by peripheral blood NK cells. In addition, NKp44 has been described to be expressed on diverse cell types, including innate lymphoid cells (ILCs) and plasmatocytoid dendritic cells (pDCs), and the binding of NKp44-expressing cells to HLA-DP might therefore result in different functional outcomes. NKp44^+^ pDCs within the tonsils for example have displayed reduced production of IFN-α after encountering an NKp44 ligand (Fuchs et al., 2005). Furthermore, there is the possibility of an inhibitory counterpart to NKp44, binding to different HLA-II molecules and inhibiting NK cell activity, similar to what has been described for activating and inhibitory KIR molecules.

LAG-3, a homolog of the CD4 molecule, has been shown to interact with HLA-II molecules (Figure 1) and is also expressed on activated NK cells (Baixeras et al., 1992; Huard et al., 1995). There are conflicting results regarding the regulation of NK cell function by LAG-3 via HLA-II. Studies in mice described an inhibition of NK cell activity after binding to HLA-DR molecules (Miyazaki et al., 1996), while later studies, using primary human NK cells, did not observe an effect on NK cell activity toward several HLA-II expressing target cell lines after blocking the LAG-3 receptor (Huard et al., 1998). Recent studies described a peptide-dependent interaction of LAG-3 with the HLA-II complex, and furthermore showed a functional inhibition of CD4^+^ T cells upon ligand engagement of LAG-3 (Maruhashi et al., 2018). Yet, an inhibition of the interaction between CD4 and HLA-II molecules by LAG-3 has not been observed (Maruhashi et al., 2018). In addition to HLA-II molecules, the liver-secreted protein fibrinogen-like protein 1 (FGL1) has been recently identified as a ligand for LAG-3 (Wang et al., 2019). FGL1 can be overexpressed on tumor cells and blocking of FGL1-LAG-3 interactions led to an increased immune activity (Wang et al., 2019). Interaction of LAG-3 with FGL1 was HLA-II-independent, indicating that LAG-3 might not be restricted to HLA-II recognition but interact with a spectrum of different cellular ligands. A second molecule described to be expressed on NK cells and to interact with HLA-II molecules is FCRL6 (Schreeder et al., 2010). FCRL6 reporter cell lines interacted with HLA-DR molecules, yet, whether FCRL6 is able to recognize a broad spectrum of HLA-DR molecules or only specific allotypes has not been determined (Schreeder et al., 2010). FCRL6 possess an intracellular immunoreceptor tyrosine-based inhibition motif (ITIM), implying that FCRL6 transmits inhibitory signaling cascades. A recent study identified high levels of FCRL6 on NK cells in HLA-II^+^ solid tumor environment, and expression of HLA-DR molecules on the surface of K562 cells inhibited the cytotoxic function of FCRL6^+^ NK-92 cells (Johnson et al., 2018). Interestingly, FCRL6 is down-modulated on the surface of NK cells upon exposure to IL-2 and IL-15 (Wilson et al., 2007), in contrast to NKp44, which expression is induced after stimulation with these cytokines. The conflicting modes of transcriptional regulation of FCRL6 and NKp44 and their opposite effects on NK cell activity might implicate FCRL6 as a potential inhibitory counterpart to NKp44 in the context of HLA-II ligand recognition (Figure 1).

NK cells are part of the innate immune system, but also play an important role in regulating adaptive as well as innate immune responses. NK cells have been shown to regulate immune cell responses of T cells (Waggoner et al., 2011; Cook et al., 2014; Crouse et al., 2015), antigen-presenting cells (Andrews et al., 2005; Moretta et al., 2005; Alter et al., 2010; Altfeld et al., 2011; Michel et al., 2012), and indirectly B cells (Rydyznski et al., 2015, 2018), all of which express HLA-II molecules. In addition, NK cells themselves are able to express HLA-II molecules (Sedlmayr et al., 1996; Erokhina et al., 2018; Costa-Garcia et al., 2019), implicating that a potential interaction between HLA-II and NK cell receptors can not only occur in *trans* but also in *cis*. The identification of HLA-II molecules as ligands for NK cell receptors now provides a possible molecular mechanism to investigate the immune cross-talk between NK cells and HLA-II-expressing immune cells, and the implications for immune responses against malignant cells and pathogens. Furthermore, a variety of non-hematopoietic cells have been described to express HLA-II molecules after exposure to IFN-γ (Kambayashi and Laufer, 2014). These “atypical” antigen-presenting cells might also represent potential targets for innate immune cell receptors recognizing HLA-II, especially under inflammatory conditions.

## HLA-II Molecules in Malignancies and Auto-Inflammatory Diseases

HLA-II molecules have been associated with the outcome of a variety of malignancies, auto-inflammatory and infectious diseases. The identification of innate immune cell receptors interacting with HLA-II now provides additional mechanisms to explain these disease associations, and can potentially lead to new therapeutic strategies. Anti-PD-1 immunotherapy has proven substantial success in the treatment of cancer patients (Page et al., 2014; Zou et al., 2016). Yet, not all patients respond to anti-PD-1 immunotherapy and some develop resistances (Kleponis et al., 2015). The level of HLA-II expression within the tumor environment can predict patient responses toward anti-PD-1 immunotherapy (Johnson et al., 2016). Interestingly, high FCRL6 expression has been detected on NK cells within HLA-II^+^ solid tumors, and blocking of FCRL6 increased the functional response of NK cells as well as T cells toward HLA-DR^+^ tumor cells (Johnson et al., 2018). In addition, FCRL6 levels were elevated at relapse within patients that progressed under anti-PD-1- therapy (Johnson et al., 2018). Therefore, the authors suggested the possibility of a combined immune checkpoint inhibitor treatment, targeting both PD-1 and FCRL6, to boost cytotoxic immune cell responses. Within certain tumors, such as colorectal carcinomas, high HLA-II expression has been associated with a favorable clinical outcome (de Bruin et al., 2008; Sconocchia et al., 2014). Induction of HLA-II expression on tumor cells has been attributed to IFN-γ exposure (de Bruin et al., 2008), indicating that the tumor microenvironment and infiltrating immune cells contribute to a favorable clinical outcome (Galon et al., 2006). However, these studies focused on T cell responses and did not exploit a possible role of innate immune cells in tumor progression. Thus, the newly identified HLA-II-NKp44 interaction might possibly contribute to the favorable prognosis of certain high HLA-II-expressing tumors.

One of the major risk factors for the development of graft-vs.-host disease (GvHD) are different HLA-DP allotypes between donor and recipient. Furthermore, in particular a single nucleotide polymorphism (SNP) within the HLA-DP β-chain that determines the expression levels of HLA-DP is associated with GvHD (Petersdorf et al., 2015), with high HLA-DP expression levels in the recipient being associated with a higher risk of developing GvHD (Petersdorf et al., 2015). The gut is one of the first sites where a GvHD response evolves, and serves as a diagnostic marker for the prognosis of GvHD. Recent studies described the expression of MHC-II molecules on the surface of intestinal epithelial cells (IECs) within the ileum of mice upon IFN-γ exposure (Koyama et al., 2019). The gut microbiota contributed to the induction of HLA-II expression, and HLA-II molecules were absent in the ileum of germ-free mice. The exposure of IECs to microbes and consequently IFN-γ secretion was essential for HLA-II expression. Interestingly, IFN-γ secretion during the course of GvHD within the murine gut was not only detected by CD4^+^ T cells but also type 1 innate lymphoid cells (ILC1s) (Koyama et al., 2019). HLA-II expression has also been described by human gut enteroid organoids after IFN-γ exposure (Koyama et al., 2019; Wosen et al., 2019), indicating that a similar mechanism might apply for the development of GvHD within humans. Which specific receptor-ligand interactions trigger IFN-γ secretion of ILC1s has to be determined, but intraepithelial IFN-γ producing ILC1s have been previously described within the tonsils and gut mucosa. Here, the secretion of IFN-γ was higher within the NKp44^+^ cell population (Fuchs et al., 2013), indicating that IFN-γ-secretion can be triggered by an NKp44-dependent mechanism.

Furthermore, specific HLA-II molecules represent risk factors for development of inflammatory bowel disease (Goyette et al., 2015). In particular, the HLA-DR β-chain HLA-DRB1^*^01:03 and the HLA-DP α-chain HLA-DPA1^*^01:03 have been associated with a higher risk of manifesting Crohn's disease. HLA-DPA1^*^01:03 interacts with a variety of HLA-DP β-chains, such as HLA-DPB1^*^04:01, to form HLA-DP401 molecules. HLA-DP401 is one of the most frequent allotypes within the Caucasoid population (Sidney et al., 2010) and interacts strongly with NKp44 (Niehrs et al., 2019). However, in patients developing Crohn's disease a reduced fraction of NKp44-expressing mucosal NK cells has been described, and high IFN-γ production contributing to disease development has been attributed to NKp46^+^ NK cells (Takayama et al., 2010), indicating an NKp44-independent mechanism. Nevertheless, ILC1s have also been shown to be high producers of IFN-γ during Crohn's disease (Bernink et al., 2013; Fuchs et al., 2013), contributing substantially to pathogenesis. Whether IFN-γ secretion by NKp44^+^ ILC1s is linked with recognition of HLA-II molecules needs to be determined. In conclusion, certain HLA-II molecules have been associated with a variety of auto-inflammatory diseases and malignancies, and NCR^+^ innate cell interactions with HLA-II molecules might provide additional molecular mechanisms underlying these disease associations.

## HLA-II Molecules in Hepatitis B Infection

Despite existence of an effective vaccine, hepatitis B virus (HBV) infection remains one of the major global health problems with more than 200 million chronically infected people (Schweitzer et al., 2015). Risk factors for the development of chronic HBV include a lack of functional Th1 cytokine responses during the acute phase of infection (Penna et al., 1997) as well as genetic factors, with SNPs within the HLA-DP region representing the main genome-wide genetic determinant for development of chronic HBV infection throughout different ethnic populations (Kamatani et al., 2009; Thomas et al., 2012). Importantly, a SNP in the 3′ untranslated region of the HLA-DP β-chain has been linked to HLA-DP surface expression levels, and HBV persistence and clearance, respectively. Low-expressed HLA-DP variants, e.g., HLA-DPB1^*^04:01 and HLA-DPB1^*^02:01, have been described to be protective while highly-expressed variants, e.g., HLA-DPB1^*^03:01 and HLA-DPB1^*^06:01, have been associated to a higher risk of developing chronic HBV (Thomas et al., 2012). The identification of binding of the NK cell receptor NKp44 to a subset of HLA-DP molecules might provide an additional molecular mechanism for the described disease association with HBV. We observed a functional interaction between NKp44 and HLA-DP401, an HLA-DP molecule associated with low surface expression and HBV clearance, but not between NKp44 and HLA-DP301, which is associated with high surface expression and HBV persistence [overview for high and low-expressed variants provided in Fleischhauer (2015)]. In a HLA-II bead-based screening assay, NKp44 interacted mainly with low-expressed HLA-DP β-chains but to some extent also displayed binding to high-expressed HLA-DP variants. Yet, we observed binding of NKp44 to high-expressed HLA-DP variants only in combination with a specific subset of HLA-DP α-chains (Niehrs et al., 2019), indicating that NKp44-binding depended on the combination of both HLA-DP chains, and suggesting that NKp44 binding occurred in close proximity to the peptide binding groove.

Interestingly, HLA-DP401 and HLA-DP201, in addition to being associated with low HLA-DP surface expression, also share a second similarity, namely the amino acid Glycine at position 84 within the HLA-DP β-chain. This amino acid position plays an important role for peptides presented by HLA-DP (Diaz et al., 2003). Recent studies have described low affinity binding of the class II invariant chain peptide (CLIP) to HLA-DP molecules possessing Gly84, and thereby insufficient blocking of the peptide binding groove of these molecules during cellular HLA-II trafficking (Yamashita et al., 2017; Anczurowski et al., 2018). Therefore, HLA-DP molecules carrying Gly84 residues are prone to present endogenous peptides derived from HLA-I peptide processing pathways (Yamashita et al., 2017). While these observations warrant further confirmation, they suggest that certain HLA-DP molecules, such as HLA-DP401 and HLA-DP201, could present intracellularly-produced peptides during HBV infection, and therefore possibly also HBV-derived peptides, which might alter the binding to TCRs of CD4^+^ T cells as well as NKp44^+^ NK cells and ILCs. Future studies characterizing the peptide-repertoires presented by HLA-II during HBV infection will provide insights into these potential interactions between NKp44^+^ immune cells and HBV-infected cells.

**Figure 2:**
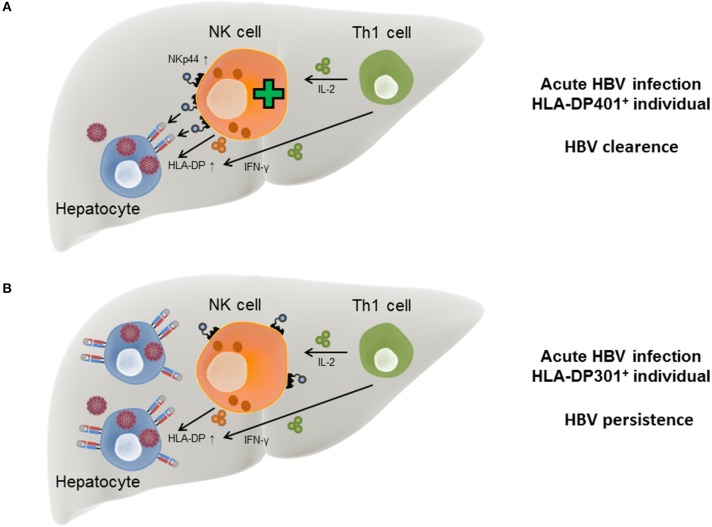
Model for HLA-DP-NKp44 interactions during HBV infection in individuals with different HLA-DP genotypes. During acute HBV infection HLA-DP molecules are upregulated on the surface of human hepatocytes in response to IFN-γ secreted by Th1 and NK cells. NKp44 expression by NK cells is initiated by IL-2 secreted by Th1 cells. In HLA-DP401^+^ HBV-infected individuals, NKp44 interacts with HLA-DP401 molecules expressed on the surface of infected hepatocytes, contributing to lysis of infected cells and HBV control **(A)**. In HLA-DP301^+^ HBV-infected individuals, NKp44 is unable to bind to HLA-DP301 molecules expressed on the surface of infected hepatocytes, resulting in inefficient lysis of infected hepatocytes by innate immune cells and higher risk of chronic HBV infection **(B)**.

In the course of acute HBV infection, IFN-γ secreted by Th1 cells has been described to be associated with self-limitation of the virus infection (Penna et al., 1997) and in addition to Th1 cells, NK cells are also able to secrete IFN-γ upon activation. During the course of HBV infection, IFN-γ has a direct anti-viral activity itself (Guidotti et al., 1999; Xia et al., 2016) but also induces HLA-II expression on hepatocytes, which in turn can function as “atypical” antigen-presenting cells (Herkel et al., 2003). The induction of an NK cell receptor ligand by pro-inflammatory cytokines is reminiscent of the induction of B7-H6, a ligand for the NKp30 receptor, upon exposure to interleukin 1-β or tumor necrosis factor-α (Matta et al., 2013). Another effector cytokine secreted by Th1 cells is interleukin-2 (IL-2), which induces the expression of NKp44 on NK cells, while NKp44 surface expression is absent on resting NK cells (Cantoni et al., 1999). These data suggest a model in which the acute phase of HBV infection induces production of IFN-γ by NK cells and Th1 cells, which also produce IL-2. IFN-γ can trigger expression of HLA-II on infected hepatocytes, while secretion of IL-2 promotes NKp44 expression on NK cells. The simultaneous expression of NKp44 and HLA-DP permits an interaction between NK cells and infected hepatocytes in individuals encoding for HLA-DP401 and other HLA-DP molecules serving as NKp44-ligands, leading to lysis of the HBV-infected cells (Figure 2A). In contrast, in HBV-infected individuals encoding for HLA-DP301 or other HLA-DP molecules not serving as NKp44-ligands, IFN-γ and IL-2 secretion can also induce HLA-DP and NKp44 expression, but NKp44^+^ cells are unable to bind to the expressed HLA-DP molecules (Figure 2B). The lack of innate immune cell recognition of HBV-infected cells might contribute to a higher risk for persistent HBV infection and provides a new mechanistic link for the described association between specific HLA-II allotypes and chronic HBV disease.

## A Possible Role for NKp44-HLA-II Interactions in NK Cell Memory

A hallmark of the innate immune system is the prompt reaction toward infected and malignant cells without the need of prior antigen-dependent stimulation. However, over the past years, studies have described an antigen-dependent memory function of NK cells (O'Leary et al., 2006; Sun et al., 2009; Paust et al., 2010). Memory NK cells have been mainly characterized by the expression of CD49a and CXCR6, are thought to be enriched in the liver and subsequently migrate to the site of infection (Paust et al., 2010; Peng et al., 2013; Reeves et al., 2015; Nikzad et al., 2019). In addition, NK cell memory has been demonstrated to be inducible by cytokine exposure as well as exposure to tumor cells (Cooper et al., 2009; Pal et al., 2017). It is still unclear which NK cell receptors mediate the observed memory responses. The newly identified interaction between NKp44 and HLA-DP was modulated by the HLA-II presented peptide, and can thus be potentially dependent on the respective antigen, indicating NKp44 as a possible receptor candidate for mediating NK cell memory responses. It is however unlikely, that NKp44 can explain all antigen-specific NK cell memory responses reported to date. Yet, NKp44-HLA-DP interactions might provide a first hint toward a possible mechanism mediating NK cell memory responses.

## Concluding Remarks

Interactions between HLA-II molecules and innate immune cells including NK cells are poorly understood. However, regulation of NK cell activity by HLA-II molecules in complex with specific pathogenic and cellular-derived peptides might help to better explain described associations between certain HLA-II allotypes and distinct outcomes of infectious as well as auto-inflammatory diseases or malignancies. Notably, the induced expression of HLA-II molecules by IFN-γ also on non-hematopoietic cells favors an interaction with NK cell receptors under inflammatory conditions. Future studies will have to determine the extent by which different NK cell receptors might interact with HLA class II molecules during physiologic conditions and in disease settings, and how these receptor-ligand interactions influence NK cell function and disease outcomes.

## Author Contributions

AN wrote the manuscript. MA revised and edited the manuscript.

### Conflict of Interest

AN and MA filed a patent application (EP18174760.1), regarding the therapeutically use of anti-NKp44 antibodies for the treatment and or prevention of graft-vs.-host disease.

## References

[B1] Alter G. Kavanagh D. Rihn S. Luteijn R. Brooks D. Oldstone M.. (2010). IL-10 induces aberrant deletion of dendritic cells by natural killer cells in the context of HIV infection. J. Clin. Invest. 120, 1905–1913. 10.1172/JCI40913 20440075 PMC2877944

[B2] Altfeld M. Fadda L. Frleta D. Bhardwaj N. (2011). DCs and NK cells, critical effectors in the immune response to HIV-1. Nat. Rev. Immunol. 11, 176–186. 10.1038/nri2935 21350578 PMC3278081

[B3] Anczurowski M. Yamashita Y. Nakatsugawa M. Ochi T. Kagoya Y. Guo T.. (2018). Mechanisms underlying the lack of endogenous processing and CLIP-mediated binding of the invariant chain by HLA-DP(84Gly). Sci. Rep. 8:4804. 10.1038/s41598-018-22931-4 29555965 PMC5859192

[B4] Andrews D. M. Andoniou C. E. Scalzo A. A. van Dommelen S. L. H. Wallace M. E. Smyth M. J.. (2005). Cross-talk between dendritic cells and natural killer cells in viral infection. Mol. Immunol. 42, 547–555. 10.1016/j.molimm.2004.07.040 15607812

[B5] Baixeras E. Huard B. Miossec C. Jitsukawa S. Martin M. Hercend T.. (1992). Characterization of the lymphocyte activation gene 3-encoded protein. A new ligand for human leukocyte antigen class II antigens. J. Exp. Med. 176, 327–337. 10.1084/jem.176.2.327 1380059 PMC2119326

[B6] Bernink J. H. Peters C. P. Munneke M. te Velde A. A. Meijer S. L. Weijer K.. (2013). Human type 1 innate lymphoid cells accumulate in inflamed mucosal tissues. Nat. Immunol. 14, 221–229. 10.1038/ni.2534 23334791

[B7] Boyington J. C. Motyka S. A. Schuck P. Brooks A. G. Sun P. D. (2000). Crystal structure of an NK cell immunoglobulin-like receptor in complex with its class I MHC ligand. Nature 405, 537–543. 10.1038/35014520 10850706

[B8] Cantoni C. Bottino C. Vitale M. Pessino A. Augugliaro R. Malaspina A.. (1999). NKp44, a triggering receptor involved in tumor cell lysis by activated human natural killer cells, is a novel member of the immunoglobulin superfamily. J. Exp. Med. 189, 787–796. 10.1084/jem.189.5.787 10049942 PMC2192947

[B9] Chapel A. Garcia-Beltran W. F. Holzemer A. Ziegler M. Lunemann S. Martrus G.. (2017). Peptide-specific engagement of the activating NK cell receptor KIR2DS1. Sci. Rep. 7:2414. 10.1038/s41598-017-02449-x 28546555 PMC5445099

[B10] Cook K. D. Waggoner S. N. Whitmire J. K. (2014). NK cells and their ability to modulate T cells during virus infections. Crit. Rev. Immunol. 34, 359–388. 10.1615/CritRevImmunol.2014010604 25404045 PMC4266186

[B11] Cooper M. A. Elliott J. M. Keyel P. A. Yang L. Carrero J. A. Yokoyama W. M. (2009). Cytokine-induced memory-like natural killer cells. Proc. Natl. Acad. Sci. U.S.A. 106, 1915–1919. 10.1073/pnas.0813192106 19181844 PMC2644138

[B12] Costa-Garcia M. Ataya M. Moraru M. Vilches C. Lopez-Botet M. Muntasell A. (2019). Human cytomegalovirus antigen presentation by HLA-DR+ NKG2C+ adaptive NK cells specifically activates polyfunctional effector memory CD4+ T lymphocytes. Front. Immunol. 10:687. 10.3389/fimmu.2019.00687 31001281 PMC6456717

[B13] Crouse J. Xu H. C. Lang P. A. Oxenius A. (2015). NK cells regulating T cell responses, mechanisms and outcome. Trends Immunol. 36, 49–58. 10.1016/j.it.2014.11.001 25432489

[B14] de Bruin E. C. van de Velde C. J. H. van Krieken J. H. J. M. Marijnen C. A. M. Medema J. P. (2008). Epithelial human leukocyte antigen-DR expression predicts reduced recurrence rates and prolonged survival in rectal cancer patients. Clin. Cancer Res. 14, 1073–1079. 10.1158/1078-0432.CCR-07-1597 18281539

[B15] Diaz G. Amicosante M. Jaraquemada D. Butler R. H. Guillen M. V. Sanchez M.. (2003). Functional analysis of HLA-DP polymorphism, a crucial role for DPbeta residues 9, 11, 35, 55, 56, 69 and 84-87 in T cell allorecognition and peptide binding. Int. Immunol. 15, 565–576. 10.1093/intimm/dxg057 12697658

[B16] Erokhina S. A. Streltsova M. A. Kanevskiy L. M. Telford W. G. Sapozhnikov A. M. Kovalenko E. I. (2018). HLA-DR(+) NK cells are mostly characterized by less mature phenotype and high functional activity. Immunol. Cell Biol. 96, 212–228. 10.1111/imcb.1032 29363179 PMC8063572

[B17] Fadda L. O'Connor G. M. Kumar S. Piechocka-Trocha A. Gardiner C. M. Carrington M.. (2011). Common HIV-1 peptide variants mediate differential binding of KIR3DL1 to HLA-Bw4 molecules. J. Virol. 85, 5970–5974. 10.1128/JVI.00412-11 21471246 PMC3126328

[B18] Fan Q. R. Long E. O. Wiley D. C. (2001). Crystal structure of the human natural killer cell inhibitory receptor KIR2DL1-HLA-Cw4 complex. Nat. Immunol. 2, 452–460. 10.1038/87766 11323700

[B19] Fleischhauer K. (2015). Immunogenetics of HLA-DP–a new view of permissible mismatches. N. Engl. J. Med. 373, 669–672. 10.1056/NEJMe1505539 26267627

[B20] Fuchs A. Cella M. Kondo T. Colonna M. (2005). Paradoxic inhibition of human natural interferon-producing cells by the activating receptor NKp44. Blood 106, 2076–2082. 10.1182/blood-2004-12-4802 15941912

[B21] Fuchs A. Vermi W. Lee J. S. Lonardi S. Gilfillan S. Newberry R. D.. (2013). Intraepithelial type 1 innate lymphoid cells are a unique subset of IL-12- and IL-15-responsive IFN-gamma-producing cells. Immunity 38, 769–781. 10.1016/j.immuni.2013.02.010 23453631 PMC3634355

[B22] Galon J. Costes A. Sanchez-Cabo F. Kirilovsky A. Mlecnik B. Lagorce-Pages C.. (2006). Type, density, and location of immune cells within human colorectal tumors predict clinical outcome. Science 313, 1960–1964. 10.1126/science.1129139 17008531

[B23] Garcia-Beltran W. F. Hölzemer A. Martrus G. Chung A. W. Pacheco Y. Simoneau C. R.. (2016). Open conformers of HLA-F are high-affinity ligands of the activating NK-cell receptor KIR3DS1. Nat. Immunol. 17, 1067–1074. 10.1038/ni.3513 27455421 PMC4992421

[B24] Goyette P. Boucher G. Mallon D. Ellinghaus E. Jostins L. Huang H.. (2015). High-density mapping of the MHC identifies a shared role for HLA-DRB1*01, 03 in inflammatory bowel diseases and heterozygous advantage in ulcerative colitis. Nat. Genet. 47, 172–179. 10.1038/ng.3176 25559196 PMC4310771

[B25] Guethlein L. A. Norman P. J. Hilton H. G. Parham P. (2015). Co-evolution of MHC class I and variable NK cell receptors in placental mammals. Immunol. Rev. 267, 259–282. 10.1111/imr.12326 26284483 PMC4587382

[B26] Guidotti L. G. Rochford R. Chung J. Shapiro M. Purcell R. Chisari F. V. (1999). Viral clearance without destruction of infected cells during acute HBV infection. Science 284, 825–829. 10.1126/science.284.5415.825 10221919

[B27] Heemskerk M. H. de Paus R. A. Lurvink E. G. Koning F. Mulder A. Willemze R.. (2001). Dual HLA class I and class II restricted recognition of alloreactive T lymphocytes mediated by a single T cell receptor complex. Proc. Natl. Acad. Sci. U.S.A. 98, 6806–6811. 10.1073/pnas.111162298 11381117 PMC34434

[B28] Herkel J. Jagemann B. Wiegard C. Lazaro J. F. G. Lueth S. Kanzler S.. (2003). MHC class II-expressing hepatocytes function as antigen-presenting cells and activate specific CD4 T lymphocyutes. Hepatology 37, 1079–1085. 10.1053/jhep.2003.50191 12717388

[B29] Holzemer A. Thobakgale C. F. Jimenez Cruz C. A. Garcia-Beltran W. F. Carlson J. M. van Teijlingen N. H.. (2015). Selection of an HLA-C^*^03, 04-restricted HIV-1 p24 Gag sequence variant is associated with viral escape from KIR2DL3+ natural killer cells, data from an observational cohort in South Africa. PLoS Med. 12:e1001900; discussion: e1001900. 10.1371/journal.pmed.1001900 26575988 PMC4648589

[B30] Huard B. Prigent P. Tournier M. Bruniquel D. Triebel F. (1995). CD4/major histocompatibility complex class II interaction analyzed with CD4- and lymphocyte activation gene-3 (LAG-3)-Ig fusion proteins. Eur. J. Immunol. 25, 2718–2721. 10.1002/eji.1830250949 7589152

[B31] Huard B. Tournier M. Triebel F. (1998). LAG-3 does not define a specific mode of natural killing in human. Immunol. Lett. 61, 109–112. 10.1016/S0165-2478(97)00170-3 9657262

[B32] Jiang Y. Z. Couriel D. Mavroudis D. A. Lewalle P. Malkovska V. Hensel N. F.. (1996). Interaction of natural killer cells with MHC class II, reversal of HLA-DR1-mediated protection of K562 transfectant from natural killer cell-mediated cytolysis by brefeldin-A. Immunology 87, 481–486. 10.1046/j.1365-2567.1996.483556.x 8778037 PMC1384120

[B33] Johnson D. B. Estrada M. V. Salgado R. Sanchez V. Doxie D. B. Opalenik S. R.. (2016). Melanoma-specific MHC-II expression represents a tumour-autonomous phenotype and predicts response to anti-PD-1/PD-L1 therapy. Nat. Commun. 7:10582. 10.1038/ncomms10582 26822383 PMC4740184

[B34] Johnson D. B. Nixon M. J. Wang Y. Wang D. Y. Castellanos E. Estrada M. V.. (2018). Tumor-specific MHC-II expression drives a unique pattern of resistance to immunotherapy via LAG-3/FCRL6 engagement. JCI Insight. 3:120360. 10.1172/jci.insight.120360 30568030 PMC6338319

[B35] Jost S. Altfeld M. (2013). Control of human viral infections by natural killer cells. Annu. Rev. Immunol. 31, 163–194. 10.1146/annurev-immunol-032712-100001 23298212

[B36] Kamatani Y. Wattanapokayakit S. Ochi H. Kawaguchi T. Takahashi A. Hosono N.. (2009). A genome-wide association study identifies variants in the HLA-DP locus associated with chronic hepatitis B in Asians. Nat. Genet. 41, 591–595. 10.1038/ng.348 19349983

[B37] Kambayashi T. Laufer T. M. (2014). Atypical MHC class II-expressing antigen-presenting cells, can anything replace a dendritic cell? Nat. Rev. Immunol. 14, 719–730. 10.1038/nri3754 25324123

[B38] Kleponis J. Skelton R. Zheng L. (2015). Fueling the engine and releasing the break, combinational therapy of cancer vaccines and immune checkpoint inhibitors. Cancer Biol. Med. 12, 201–208. 10.7497/j.issn.2095-3941.2015.0046 26487965 PMC4607816

[B39] Koyama M. Mukhopadhyay P. Schuster I. S. Henden A. S. Hulsdunker J. Varelias A.. (2019). MHC class II antigen presentation by the intestinal epithelium initiates graft-versus-host disease and is influenced by the microbiota. Immunity. 51, 885–98.e7 10.1016/j.immuni.2019.08.011 31542340 PMC6959419

[B40] Liu J. Xiao Z. Ko H. L. Shen M. Ren E. C. (2014). Activating killer cell immunoglobulin-like receptor 2DS2 binds to HLA-A^*^11. Proc. Natl. Acad. Sci. U.S.A. 111, 2662–2667. 10.1073/pnas.1322052111 24550293 PMC3932910

[B41] Lobo P. I. Chang M. Y. Mellins E. (1996). Mechanisms by which HLA-class II molecules protect human B lymphoid tumour cells against NK- and LAK-mediated cytolysis. Immunology 88, 625–629. 10.1046/j.1365-2567.1996.d01-679.x 8881767 PMC1456640

[B42] Maenaka K. Juji T. Stuart D. I. Jones E. Y. (1999). Crystal structure of the human p58 killer cell inhibitory receptor (KIR2DL3) specific for HLA-Cw3-related MHC class I. Structure 7, 391–398. 10.1016/S0969-2126(99)80052-5 10196125

[B43] Maruhashi T. Okazaki I.-M. Sugiura D. Takahashi S. Maeda T. K. Shimizu K.. (2018). LAG-3 inhibits the activation of CD4(+) T cells that recognize stable pMHCII through its conformation-dependent recognition of pMHCII. Nat. Immunol. 19, 1415–1426. 10.1038/s41590-018-0217-9 30349037

[B44] Matta J. Baratin M. Chiche L. Forel J.-M. Cognet C. Thomas G.. (2013). Induction of B7-H6, a ligand for the natural killer cell-activating receptor NKp30, in inflammatory conditions. Blood 122, 394–404. 10.1182/blood-2013-01-481705 23687088

[B45] Michel T. Hentges F. Zimmer J. (2012). Consequences of the crosstalk between monocytes/macrophages and natural killer cells. Front. Immunol. 3:403. 10.3389/fimmu.2012.00403 23316194 PMC3539656

[B46] Miyazaki T. Dierich A. Benoist C. Mathis D. (1996). Independent modes of natural killing distinguished in mice lacking Lag3. Science 272, 405–408. 10.1126/science.272.5260.405 8602528

[B47] Moesta A. K. Norman P. J. Yawata M. Yawata N. Gleimer M. Parham P. (2008). Synergistic polymorphism at two positions distal to the ligand-binding site makes KIR2DL2 a stronger receptor for HLA-C than KIR2DL3. J. Immunol. 180, 3969–3979. 10.4049/jimmunol.180.6.3969 18322206

[B48] Moretta A. Marcenaro E. Sivori S. Della Chiesa M. Vitale M. Moretta L. (2005). Early liaisons between cells of the innate immune system in inflamed peripheral tissues. Trends Immunol. 26, 668–675. 10.1016/j.it.2005.09.008 16198147

[B49] Muhlethaler-Mottet A. Otten L. A. Steimle V. Mach B. (1997). Expression of MHC class II molecules in different cellular and functional compartments is controlled by differential usage of multiple promoters of the transactivator CIITA. EMBO J. 16, 2851–2860. 10.1093/emboj/16.10.2851 9184229 PMC1169893

[B50] Naiyer M. M. Cassidy S. A. Magri A. Cowton V. Chen K. Mansour S.. (2017). KIR2DS2 recognizes conserved peptides derived from viral helicases in the context of HLA-C. Sci. Immunol. 2:eaal5296. 10.1126/sciimmunol.aal5296 28916719

[B51] Niehrs A. Garcia-Beltran W. F. Norman P. J. Watson G. M. Hölzemer A. Chapel A.. (2019). A subset of HLA-DP molecules serve as ligands for the natural cytotoxicity receptor NKp44. Nat. Immunol. 20, 1129–37. 10.1038/s41590-019-0448-4 31358998 PMC8370669

[B52] Nikzad R. Angelo L. S. Aviles-Padilla K. Le D. T. Singh V. K. Bimler L.. (2019). Human natural killer cells mediate adaptive immunity to viral antigens. Sci. Immunol. 4:eaat8116. 10.1126/sciimmunol.aat8116 31076527 PMC6636344

[B53] O'Connor G. M. Vivian J. P. Gostick E. Pymm P. Lafont B. A. P. Price D. A.. (2015). Peptide-dependent recognition of HLA-B^*^57, 01 by KIR3DS1. J. Virol. 89, 5213–5221. 10.1128/JVI.03586-14 25740999 PMC4442525

[B54] O'Leary J. G. Goodarzi M. Drayton D. L. von Andrian U. H. (2006). T cell- and B cell-independent adaptive immunity mediated by natural killer cells. Nat. Immunol. 7, 507–516. 10.1038/ni1332 16617337

[B55] Page D. B. Postow M. A. Callahan M. K. Allison J. P. Wolchok J. D. (2014). Immune modulation in cancer with antibodies. Annu. Rev. Med. 65, 185–202. 10.1146/annurev-med-092012-112807 24188664

[B56] Pal M. Schwab L. Yermakova A. Mace E. M. Claus R. Krahl A.-C.. (2017). Tumor-priming converts NK cells to memory-like NK cells. Oncoimmunology 6:e1317411. 10.1080/2162402X.2017.1317411 28680749 PMC5486172

[B57] Paust S. Gill H. S. Wang B.-Z. Flynn M. P. Moseman E. A. Senman B.. (2010). Critical role for the chemokine receptor CXCR6 in NK cell-mediated antigen-specific memory of haptens and viruses. Nat. Immunol. 11, 1127–1135. 10.1038/ni.1953 20972432 PMC2982944

[B58] Peng H. Jiang X. Chen Y. Sojka D. K. Wei H. Gao X.. (2013). Liver-resident NK cells confer adaptive immunity in skin-contact inflammation. J. Clin. Invest. 123, 1444–1456. 10.1172/JCI66381 23524967 PMC3613925

[B59] Penna A. Del Prete G. Cavalli A. Bertoletti A. D'Elios M. M. Sorrentino R.. (1997). Predominant T-helper 1 cytokine profile of hepatitis B virus nucleocapsid-specific T cells in acute self-limited hepatitis B. Hepatology 25, 1022–1027. 10.1002/hep.510250438 9096614

[B60] Petersdorf E. W. Malkki M. O'hUigin C. Carrington M. Gooley T. Haagenson M. D.. (2015). High HLA-DP expression and graft-versus-host disease. N. Engl. J. Med. 373, 599–609. 10.1056/NEJMoa1500140 26267621 PMC4560117

[B61] Rahim M. M. A. Tu M. M. Mahmoud A. B. Wight A. Abou-Samra E. Lima P. D. A.. (2014). Ly49 receptors, innate and adaptive immune paradigms. Front. Immunol. 5:145. 10.3389/fimmu.2014.00145 24765094 PMC3980100

[B62] Ranasinghe S. Lamothe P. A. Soghoian D. Z. Kazer S. W. Cole M. B. Shalek A. K.. (2016). Antiviral CD8+T cells restricted by human leukocyte antigen class II exist during natural HIV infection and exhibit clonal expansion. Immunity 45, 917–930. 10.1016/j.immuni.2016.09.015 27760342 PMC5077698

[B63] Reeves R. K. Li H. Jost S. Blass E. Li H. Schafer J. L.. (2015). Antigen-specific NK cell memory in rhesus macaques. Nat. Immunol. 16, 927–932. 10.1038/ni.3227 26193080 PMC4545390

[B64] Rist M. Smith C. Bell M. J. Burrows S. R. Khanna R. (2009). Cross-recognition of HLA DR4 alloantigen by virus-specific CD8+ T cells, a new paradigm for self-/nonself-recognition. Blood 114, 2244–2253. 10.1182/blood-2009-05-222596 19617574

[B65] Rydyznski C. Daniels K. A. Karmele E. P. Brooks T. R. Mahl S. E. Moran M. T.. (2015). Generation of cellular immune memory and B-cell immunity is impaired by natural killer cells. Nat. Commun. 6:6375. 10.1038/ncomms7375 25721802 PMC4346304

[B66] Rydyznski C. E. Cranert S. A. Zhou J. Q. Xu H. Kleinstein S. H. Singh H.. (2018). Affinity maturation is impaired by natural killer cell suppression of germinal centers. Cell Rep. 24, 3367–3373.e4. 10.1016/j.celrep.2018.08.075 30257198 PMC6192537

[B67] Schreeder D. M. Cannon J. P. Wu J. Li R. Shakhmatov M. A. Davis R. S. (2010). Cutting edge, FcR-Like 6 is an MHC class II receptor. J. Immunol. 185, 23–27. 10.4049/jimmunol.1000832 20519654 PMC4876727

[B68] Schweitzer A. Horn J. Mikolajczyk R. T. Krause G. Ott J. J. (2015). Estimations of worldwide prevalence of chronic hepatitis B virus infection, a systematic review of data published between 1965 and 2013. Lancet (London, England). 386, 1546–1555. 10.1016/S0140-6736(15)61412-X 26231459

[B69] Sconocchia G. Eppenberger-Castori S. Zlobec I. Karamitopoulou E. Arriga R. Coppola A.. (2014). HLA class II antigen expression in colorectal carcinoma tumors as a favorable prognostic marker. Neoplasia 16, 31–42. 10.1593/neo.131568 24563618 PMC3924546

[B70] Sedlmayr P. Schallhammer L. Hammer A. Wilders-Truschnig M. Wintersteiger R. Dohr G. (1996). Differential phenotypic properties of human peripheral blood CD56dim+ and CD56bright+ natural killer cell subpopulations. Int. Arch. Allergy Immunol. 110, 308–313. 10.1159/000237321 8768796

[B71] Sidney J. Steen A. Moore C. Ngo S. Chung J. Peters B.. (2010). Five HLA-DP molecules frequently expressed in the worldwide human population share a common HLA supertypic binding specificity. J. Immunol. 184, 2492–2503. 10.4049/jimmunol.0903655 20139279 PMC2935290

[B72] Siewiera J. Gouilly J. Hocine H.-R. Cartron G. Levy C. Al-Daccak R.. (2015). Natural cytotoxicity receptor splice variants orchestrate the distinct functions of human natural killer cell subtypes. Nat. Commun. 6:10183. 10.1038/ncomms10183 26666685 PMC4682172

[B73] Stevanovic S. van Bergen C. A. M. van Luxemburg-Hejis S. A. P. van der Zouwen B. Jordanova E. S. Kruisselbrink A. B. (2013). HLA class II upregulation during viral infection leads to HLA-DP – directed graft-versus-host disease after CD4 1 donor lymphocyte infusion. Am. Soc. Hematol. 122, 1963–1974. 10.1182/blood-2012-12-470872 23777765

[B74] Sun J. C. Beilke J. N. Lanier L. L. (2009). Adaptive immune features of natural killer cells. Nature 457, 557–561. 10.1038/nature07665 19136945 PMC2674434

[B75] Takayama T. Kamada N. Chinen H. Okamoto S. Kitazume M. T. Chang J.. (2010). Imbalance of NKp44(+)NKp46(-) and NKp44(-)NKp46(+) natural killer cells in the intestinal mucosa of patients with Crohn's disease. Gastroenterology 139, 882–892, 892.e1–3. 10.1053/j.gastro.2010.05.040 20638936

[B76] Thomas R. Thio C. L. Apps R. Qi Y. Gao X. Marti D.. (2012). A novel variant marking HLA-DP expression levels predicts recovery from hepatitis B virus infection. J. Virol. 86, 6979–6985. 10.1128/JVI.00406-12 22496224 PMC3393572

[B77] Ting J. P.-Y. Trowsdale J. (2002). Genetic control of MHC class II expression. Cell 109(Suppl), S21–33. 10.1016/S0092-8674(02)00696-7 11983150

[B78] Vales-Gomez M. Reyburn H. T. Erskine R. A. Strominger J. (1998). Differential binding to HLA-C of p50-activating and p58-inhibitory natural killer cell receptors. Proc. Natl. Acad. Sci. U.S.A. 95, 14326–14331. 10.1073/pnas.95.24.14326 9826699 PMC24372

[B79] Waggoner S. N. Cornberg M. Selin L. K. Welsh R. M. (2011). Natural killer cells act as rheostats modulating antiviral T cells. Nature 481, 394–398. 10.1038/nature10624 22101430 PMC3539796

[B80] Wang J. Sanmamed M. F. Datar I. Su T. T. Ji L. Sun J.. (2019). Fibrinogen-like protein 1 is a major immune inhibitory ligand of LAG-3. Cell 176, 334–347.e12. 10.1016/j.cell.2018.11.010 30580966 PMC6365968

[B81] Wilson T. J. Presti R. M. Tassi I. Overton E. T. Cella M. Colonna M. (2007). FcRL6, a new ITIM-bearing receptor on cytolytic cells, is broadly expressed by lymphocytes following HIV-1 infection. Blood 109, 3786–3793. 10.1182/blood-2006-06-030023 17213291

[B82] Wosen J. E. Ilstad-Minnihan A. Co J. Y. Jiang W. Mukhopadhyay D. Fernandez-Becker N. Q.. (2019). Human intestinal enteroids model MHC-II in the gut epithelium. Front. Immunol. 10:1970. 10.3389/fimmu.2019.01970 31481960 PMC6710476

[B83] Xia Y. Stadler D. Lucifora J. Reisinger F. Webb D. Hosel M.. (2016). Interferon-gamma and tumor necrosis factor-alpha produced by T cells reduce the HBV persistence form, cccDNA, without cytolysis. Gastroenterology 150, 194–205. 10.1053/j.gastro.2015.09.026 26416327

[B84] Yamashita Y. Anczurowski M. Nakatsugawa M. Tanaka M. Kagoya Y. Sinha A.. (2017). HLA-DP(84Gly) constitutively presents endogenous peptides generated by the class I antigen processing pathway. Nat. Commun. 8:15244. 10.1038/ncomms15244 28489076 PMC5436232

[B85] Zou W. Wolchok J. D. Chen L. (2016). PD-L1 (B7-H1) and PD-1 pathway blockade for cancer therapy, Mechanisms, response biomarkers, and combinations. Sci. Transl. Med. 8:328rv4. 10.1126/scitranslmed.aad7118 26936508 PMC4859220

